# Supplementary material for: Identification of photoperiod-induced specific miRNAs in the adrenal glands of Sunite sheep (Ovis aries)
Source: Front Vet Sci. 2022 Jul 22;9:888207. doi: 10.3389/fvets.2022.888207 (PMC9354845; doi:10.3389/fvets.2022.888207)
Supplement: Supplementary file 1 [file Data_Sheet_1.ZIP › Supplementary file/miRDeep2 file.zip!/miRDeep2 file/Novel_Structure/SP42A2/Novel_422.pdf]

Provisional ID	:	NC_019460.2_9314
Score total	:	2.6
Score for star read(s)	:	-1.3
Score for read counts	:	0
Score for mfe	:	2.3
Score for randfold	:	1.6
Score for cons. seed	:	
Total read count	:	75
Mature read count	:	75
Loop read count	:	0
Star read count	:	0

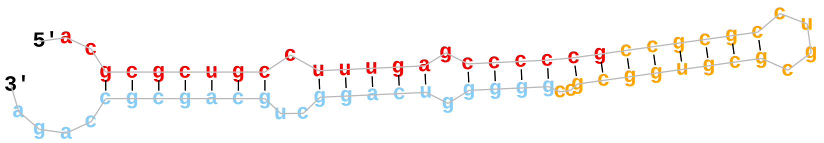

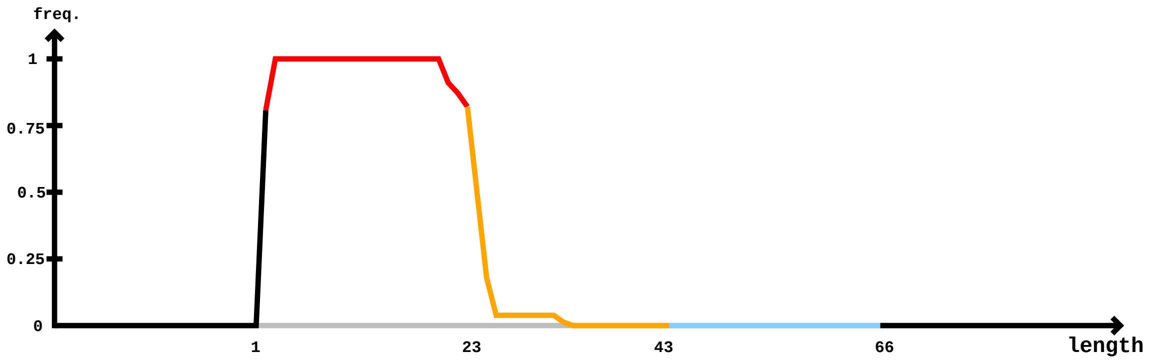

Mature Star

```
5'- ccaaucuaaaugucugaugaacgcgcugccuuugagcccccgccgcgccugcgcguggcgccgggggucaggcugcagcgccagaggacaugcucgcgcauaacugggg   -3'    exp
    (((......((((((..((...(((((((.(((((.((((((((((((....))))))))..)))).)))))..)))))))))..))))))((....)).....)))..    reads      mm          sample
    ....................acgcgcugccuuugagccc......................................................................    6          0           seq
    ....................acgcgcugccuuugagcccc.....................................................................    2          0           seq
    ....................acgcgcugccuuugagccccc....................................................................    3          0           seq
    ....................acgcgcugccuuugagcccccg...................................................................    32         0           seq
    ....................acgcgcugccuuugagcccccgc..................................................................    11         0           seq
    ....................acgcgcugccuuugagcccccgcc.................................................................    6          0           seq
    ....................acgcgcugccuuugagcccccgccgcgccug..........................................................    2          0           seq
    ....................acgcgcugccuuugagcccccgccgcgccugc.........................................................    1          0           seq
    .....................cgcgcugccuuugagccc......................................................................    1          0           seq
    .....................cgcgcugccuuugagcccc.....................................................................    1          0           seq
    .....................cgcgcugccuuugagccccc....................................................................    1          0           seq
    .....................cgcgcugccuuugagcccccg...................................................................    5          0           seq
    .....................cgcgcugccuuugagcccccgc..................................................................    2          0           seq
    .....................cgcgcugccuuugagcccccgcc.................................................................    5          0           seq
```
